# Estimates of Excess Medically Attended Acute Respiratory Infections in Periods of Seasonal and Pandemic Influenza in Germany from 2001/02 to 2010/11

**DOI:** 10.1371/journal.pone.0064593

**Published:** 2013-07-16

**Authors:** Matthias an der Heiden, Karla Köpke, Silke Buda, Udo Buchholz, Walter Haas

**Affiliations:** Department of Infectious Disease Epidemiology, Robert Koch Institute, Berlin, Germany; University of Hong Kong, Hong Kong

## Abstract

**Background:**

The number of patients seeking health care is a central indicator that may serve several different purposes: (1) as a proxy for the impact on the burden of the primary care system; (2) as a starting point to estimate the number of persons ill with influenza; (3) as the denominator data for the calculation of case fatality rate and the proportion hospitalized (severity indicators); (4) for economic calculations. In addition, reliable estimates of burden of disease and on the health care system are essential to communicate the impact of influenza to health care professionals, public health professionals and to the public.

**Methodology/Principal Findings:**

Using German syndromic surveillance data, we have developed a novel approach to describe the seasonal variation of medically attended acute respiratory infections (MAARI) and estimate the excess MAARI attributable to influenza. The weekly excess inside a period of influenza circulation is estimated as the difference between the actual MAARI and a MAARI-baseline, which is established using a cyclic regression model for counts. As a result, we estimated the highest ARI burden within the last 10 years for the influenza season 2004/05 with an excess of 7.5 million outpatient visits (CI95% 6.8–8.0). In contrast, the pandemic wave 2009 accounted for one third of this burden with an excess of 2.4 million (CI95% 1.9–2.8). Estimates can be produced for different age groups, different geographic regions in Germany and also in real time during the influenza waves.

## Introduction

In the context of the course of the influenza pandemic (H1N1) 2009 it became clear, that it is of paramount importance to be able to provide estimates of the burden of disease in the population and the severity of the disease. The number of patients seeking health care is a central indicator that may serve several different purposes: (1) as a proxy for the impact on the burden of the primary care system; (2) as a starting point to estimate the number of persons ill with influenza; (3) as the denominator data for the calculation of case fatality rate and the proportion hospitalized (severity indicators); (4) for economic calculations. In addition, reliable estimates of burden of disease and on the health care system are essential to communicate the impact of influenza to health care professionals, public health professionals and to the public. As an influenza epidemic unfolds it is important to obtain an estimate of the burden on the health care system that is both timely and that can be updated for example on a weekly basis. This information is a prerequisite to give sound advice for political decisions.

To estimate the impact of influenza on the health care system we used data of the German syndromic surveillance system for influenza that counts cases of medically attended acute respiratory illness (MAARI). Representative data are available since October 2001. The large and dynamic background of MAARI complicates the estimation of the proportion that is attributable to influenza. The objective of this paper was to develop a standard method that is capable (1) to estimate retrospectively the overall burden of influenza-associated MAARI for the epidemic waves of the previous 10 years (2001/02 to 2010/11), and (2) to prospectively estimate the weekly number of MAARI attributable to influenza.

## Results

### Participation and reporting of MAARI

**Figure 1:**
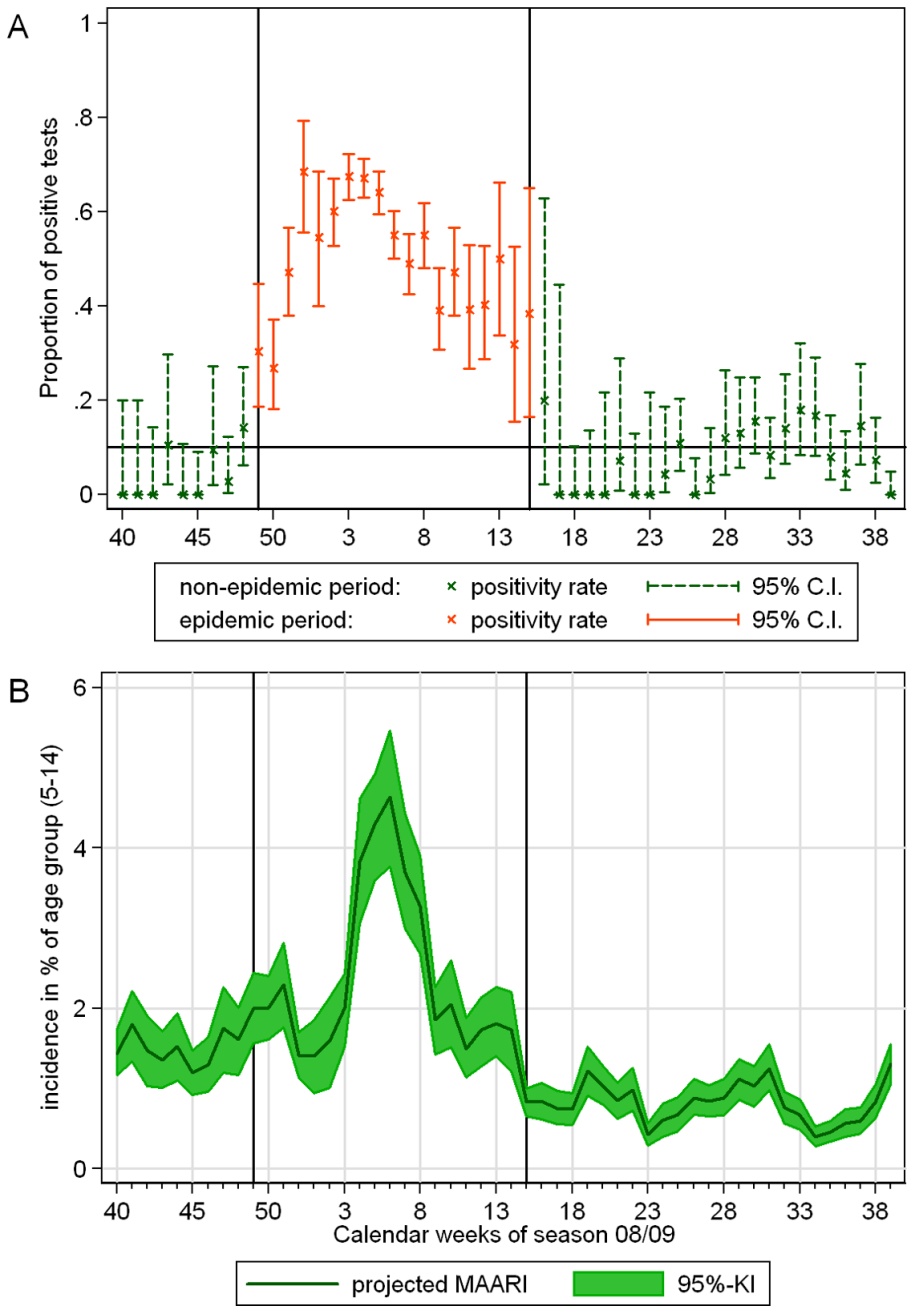
Course of positivity rate and MAARI in season 2008/09. (A) Positivity rate of influenza in samples investigated by the NRCI. Weeks of the epidemic period are colored red, whereas all other weeks are green; (B) MAARI incidence in percent of the population of the southern region, in age group (15–34); the black horizontal lines indicate the beginning and end of the epidemic period.

First of all, we briefly describe the participation and reporting of MAARI of physicians in the AGI system. On average 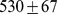 AGI physicians reported per week in the summer seasons between 2006 and 2011, whereas on average 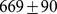 physicians reported per week in the winter seasons between 2001/02 and 2010/11. The average number of physicians by specialty and region in Germany over the years 2001 to 2010 can be seen in Table S1 of File S1 in the supporting information. An example for the course of the projected MAARI can be seen in Figure 1.

### Periods of influenza circulation

Using the definitions given in section Materials and Methods a single epidemic period could be assigned to each season analyzed, including the pandemic season 2009/10, (see Figure 1 and Table 1). The epidemic period began always shortly before or after the turn of the year, only in season 2005/06 it started in February, see Table 0. In contrast, in the pandemic season 2009/10 the PIC started already in October in Germany. Cases of pandemic influenza A(H1N1)pdm2009 were observed in Germany since April 2009, but during the summer season 2009 we detected no epidemic.

**Table 1:** Begin and end of the period of influenza circulation per season based on virological data collected by the NRCI, Germany.

season	begin	end	duration [in weeks]
2001/02	2002 w 04	2002 w 15	12
2002/03	2003 w 04	2003 w 15	12
2003/04	2003 w 51	2004 w 14	16
2004/05	2005 w 02	2005 w 14	13
2005/06	2006 w 07	2006 w 17	11
2006/07	2007 w 02	2007 w 15	14
2007/08	2007 w 52	2008 w 17	18
2008/09	2008 w 49	2009 w 15	19
2009/10	2009 w 42	2010 w 04	16
2010/11	2010 w 50	2011 w 14	17

### Estimation of the MAARI baseline

The analysis of the estimated number of MAARI in Germany aggregated over the regions – outside of epidemic periods and the 2 weeks around the turn of the year – revealed that a cubic trend described the data most appropriately. Regarding the annual pattern, we found that the first 5 overtones to the annual oscillation improved the model fit significantly, whereas the sine and cosine with 6 periods per year failed to further improve the model fit. The sine and cosine functions with a period of 2 up to 5 years significantly improved the model.

**Figure 2:**
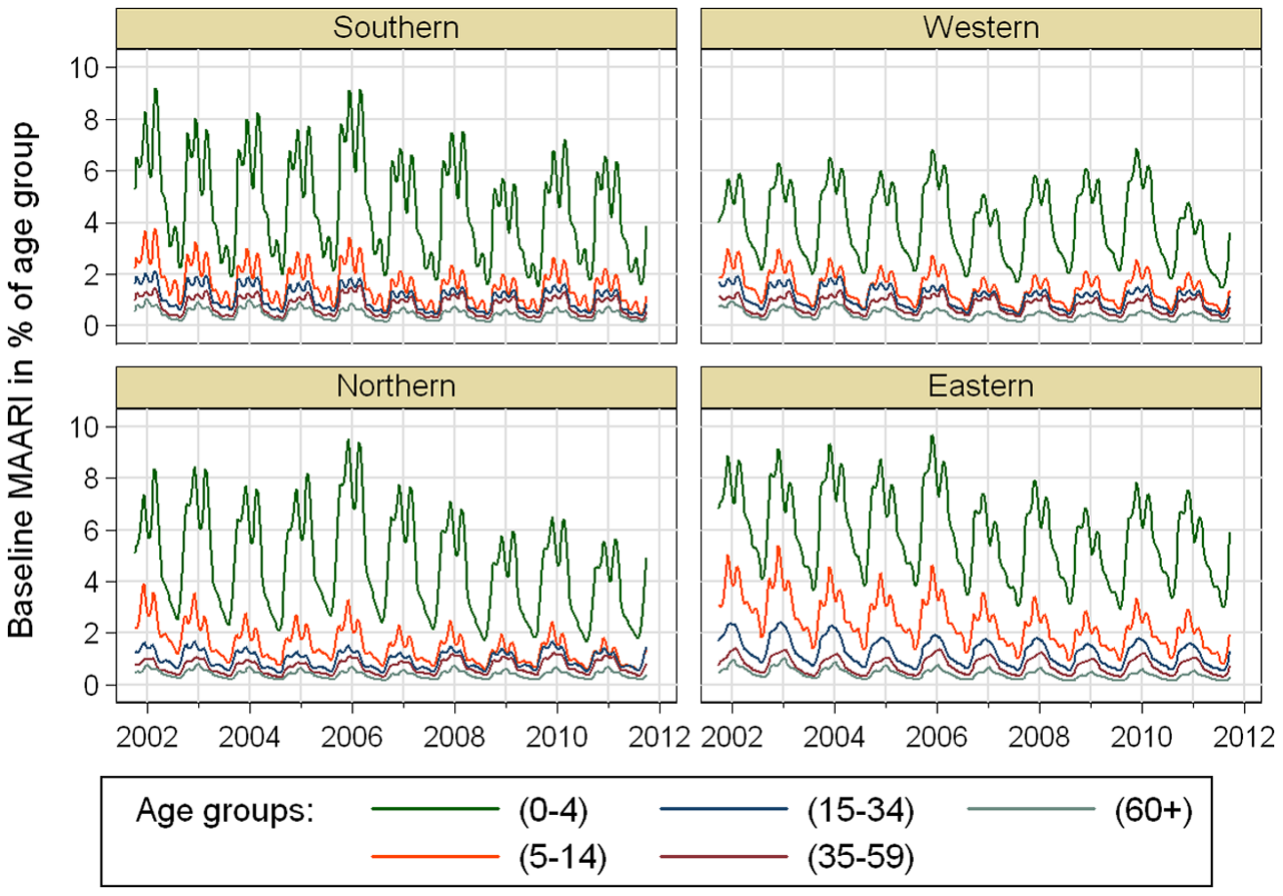
Estimated MAARI baselines in different age groups for regions of Germany.

The MAARI baselines for the different age groups were clearly separated from each other (see Figure 2). Moreover, the effect of age group was significantly modified by region. The MAARI baseline for children of age group (5–14) was particularly high in the Eastern region. Nearly all baselines showed a downward peak shortly after the turn of the year and reached their minimum in summer around the time of the school holidays.

### Retrospective estimation of excess MAARI

**Figure 3:**
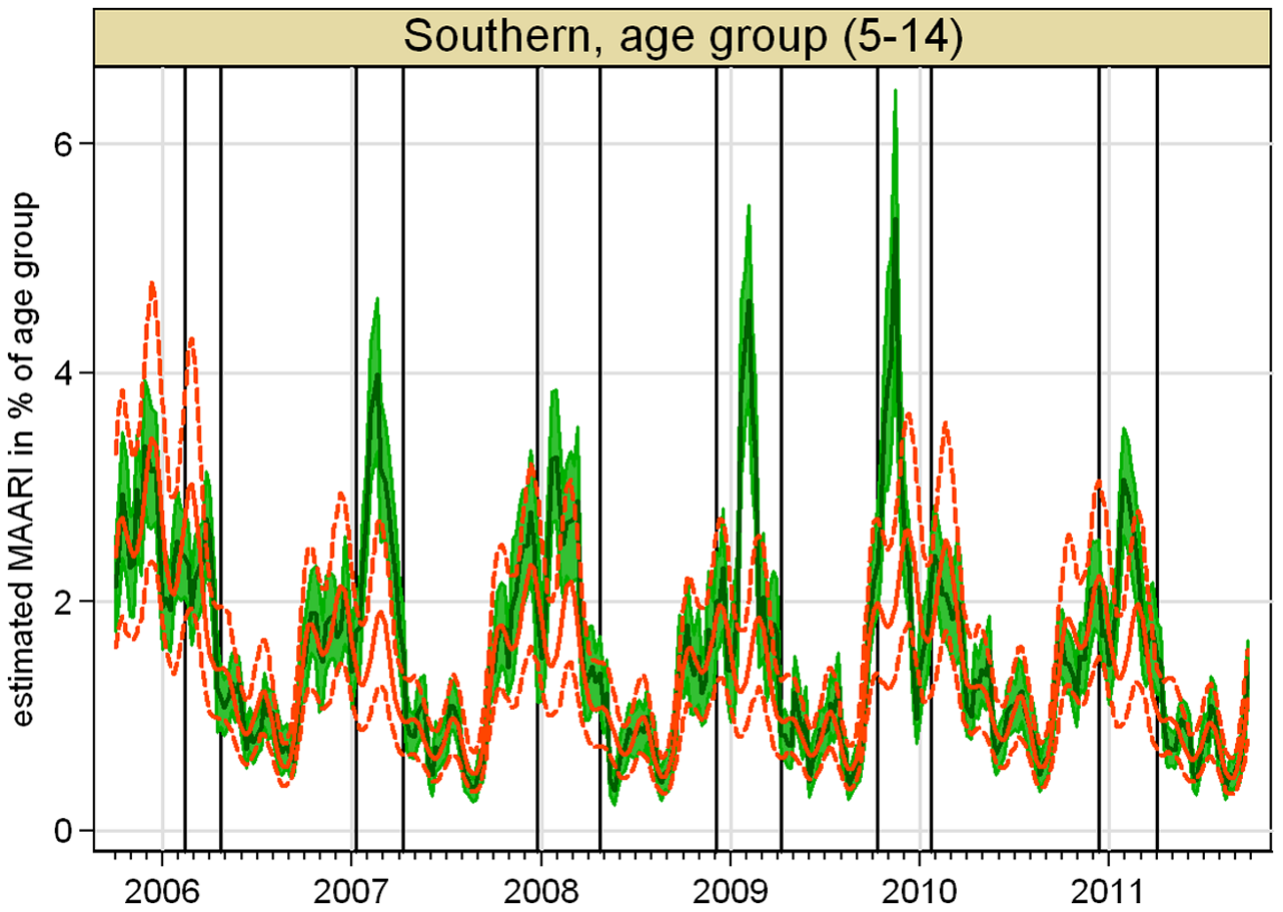
Projected MAARI (darkgreen) with 95% confidence interval (green) and estimated MAARI baseline (red) with 95% prediction interval (dotted red line) for age group (5–14) in the southern region, starting from season 2006/07, vertical lines indicate beginning and end of epidemic periods.

An example for a regional baseline together with 95% upper and lower prediction limits and the projected number of MAARI for years with summer surveillance data is shown in Figure 3. Further figures are shown in the supporting information.

**Figure 4:**
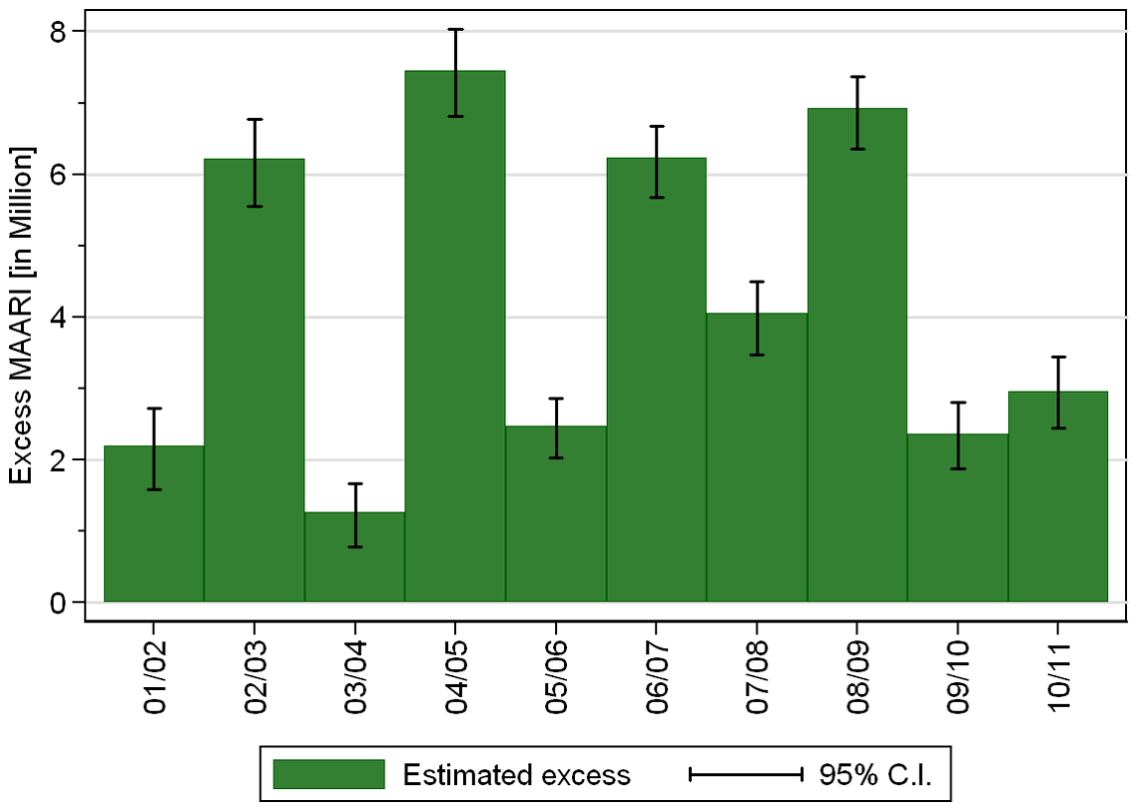
Estimated total excess MAARI inside epidemic periods.

We found in all investigated seasons a significant total (positive) excess of MAARI during the epidemic period (Figure 4 and Table 2). The total negative excess during the epidemic periods was always considerably smaller. On the other hand, the total positive and negative excesses were of the same magnitude for non-epidemic periods.

**Table 2:** Estimated total positive and negative excess MAARI [in Million] in epidemic and non-epidemic periods of influenza.

	total excess MAARI		total negative excess MAARI	
season	epidemic period	non-epidemic period	epidemic period	non-epidemic period
2001/02	2.2 (1.6, 2.7)	0.0 (−0.4, 0.4)	−0.2 (−0.4, 0.0)	−0.1 (−0.6, 0.2)
2002/03	6.2 (5.6, 6.8)	0.1 (−0.3, 0.5)	0.0 (0.0, 0.0)	−0.1 (−0.5, 0.3)
2003/04	1.3 (0.8, 1.7)	0.1 (−0.3, 0.4)	−0.2 (−0.6, 0.2)	−0.1 (−0.5, 0.3)
2004/05	7.5 (6.8, 8.0)	0.1 (−0.2, 0.3)	0.0 (0.0, 0.0)	−0.1 (−0.5, 0.3)
2005/06	2.5 (2.0, 2.9)	0.1 (−0.4, 0.5)	−0.1 (−0.4, 0.1)	−0.1 (−0.7, 0.4)
2006/07	6.2 (5.7, 6.7)	0.1 (−0.1, 0.2)	0.0 (0.0, 0.0)	−0.5 (−1.0, 0.0)
2007/08	4.1 (3.5, 4.5)	0.5 (0.1, 0.9)	0.0 (0.0, 0.0)	−0.1 (−0.4, 0.2)
2008/09	6.9 (6.4, 7.4)	0.2 (−0.2, 0.5)	0.0 (0.0, 0.0)	−0.2 (−0.5, 0.1)
2009/10	2.4 (1.9, 2.8)	0.6 (0.1, 1.1)	−0.2 (−0.4, −0.1)	0.0 (−0.1, 0.1)
2010/11	3.0 (2.4, 3.4)	0.0 (−0.1, 0.1)	0.0 (−0.2, 0.1)	−0.6 (−1.0, −0.2)

Between 2001/02 and 2008/09 the total excess MAARI for Germany averaged 4.3 million per season and ranged from 1.3 to 7.5 million. During the 2009 pandemic A(H1N1) season the estimated total excess MAARI was 2.4 million (Table 2).

**Figure 5:**
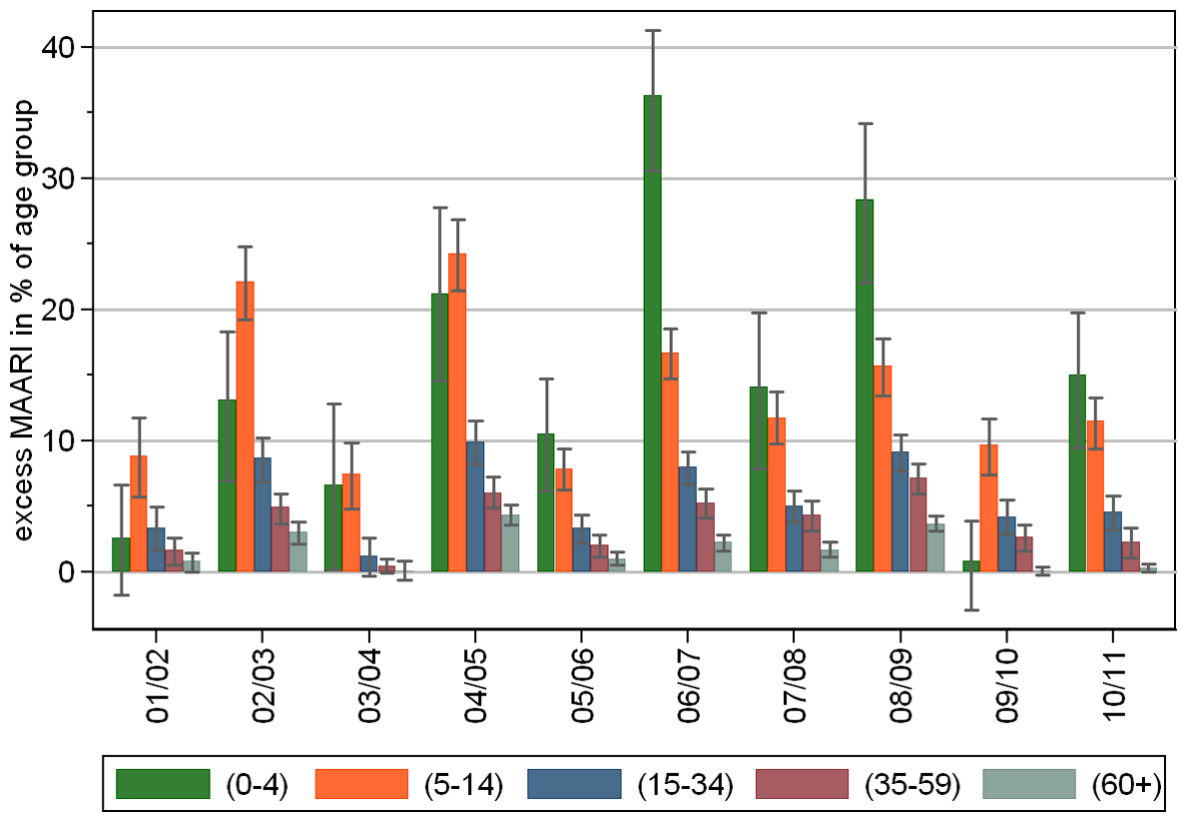
Estimated total excess MAARI inside epidemic periods as percentage of the population by season and age group.

Among the different age groups the total excess MAARI in percent of the population ranged inside epidemic periods between around 1% of persons in age group (60+) and up to 36% of children in the age group (0–4), see Figure 5. Expressed differently, among 10 seasons a total excess MAARI incidence of 5% was exceeded in all seasons by children in the age group (5–14), by children aged 0–4 years 8 out of 10 times, by persons aged 15–34 years 5 out of 10 times, by persons aged 35–59 years only 3 out of 10 times, and never by persons aged at least 60 years.

In all seasons including the pandemic 2009/10 the incidence of excess MAARI in the age groups above 4 years showed a tendency to decrease with age. In 6 of the 10 seasons children aged 0 to 4 years had the highest incidence and in 3 the second highest (Figure 5).

**Figure 6:**
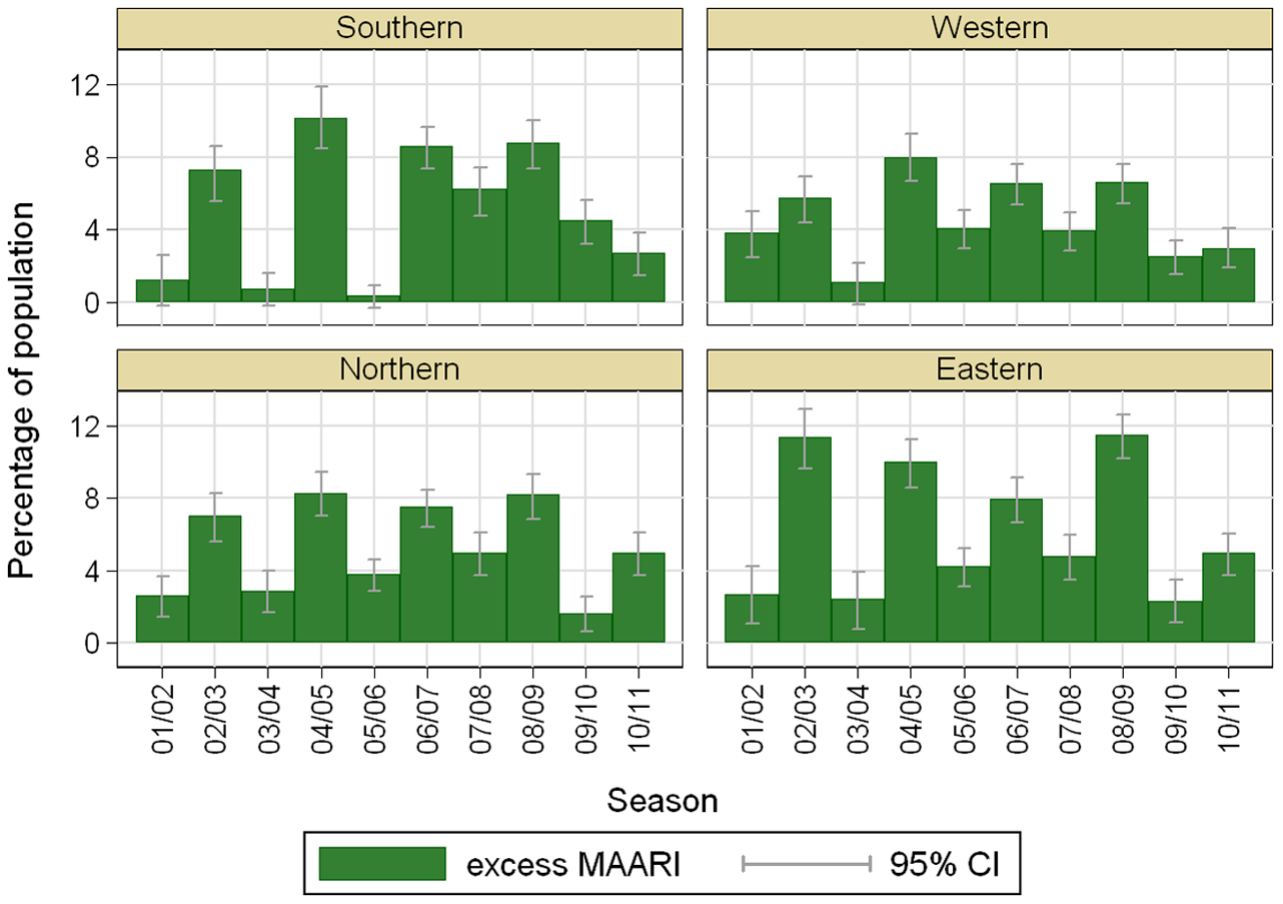
Estimated total excess MAARI inside epidemic periods as percentage of the population by season and region.

The estimated total excess MAARI showed different pattern for the different region showing that the relative strength of the seasons differs between the regions, see Figure 6.

### Sensitivity analysis

**Figure 7:**
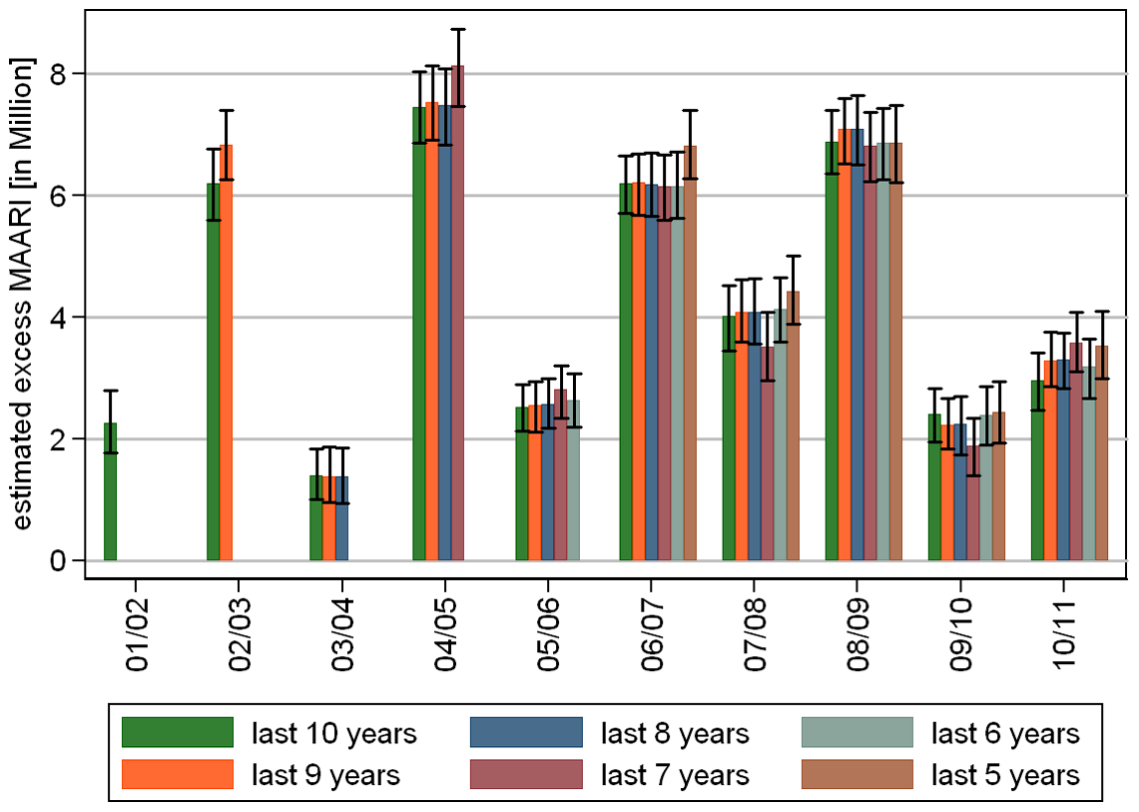
(Sensitivity analysis 1) Comparison of estimated total excess MAARI during epidemic periods depending on the number of seasons used retrospectively to estimate the MAARI baseline.

Sensitivity analysis with data sets including the most recent 5 to 10 seasons led to the following results: The model selection step resulted always in 5 overtones to the annual oscillation as well as oscillations with periods of up to half the length of the examination period. The polynomial trend had degree 2 for the data set (2001/02 – 2006/07) and (2001/02 – 2007/08), and degree 3 in all other cases. The resulting model fit showed no substantial variation in the estimated total excess MAARI during epidemic periods when the baseline was built on fewer and the more recent seasons, see Figure 7. Seasons lying on the boundary of the chosen investigation period showed in some cases a higher estimate.

**Figure 8:**
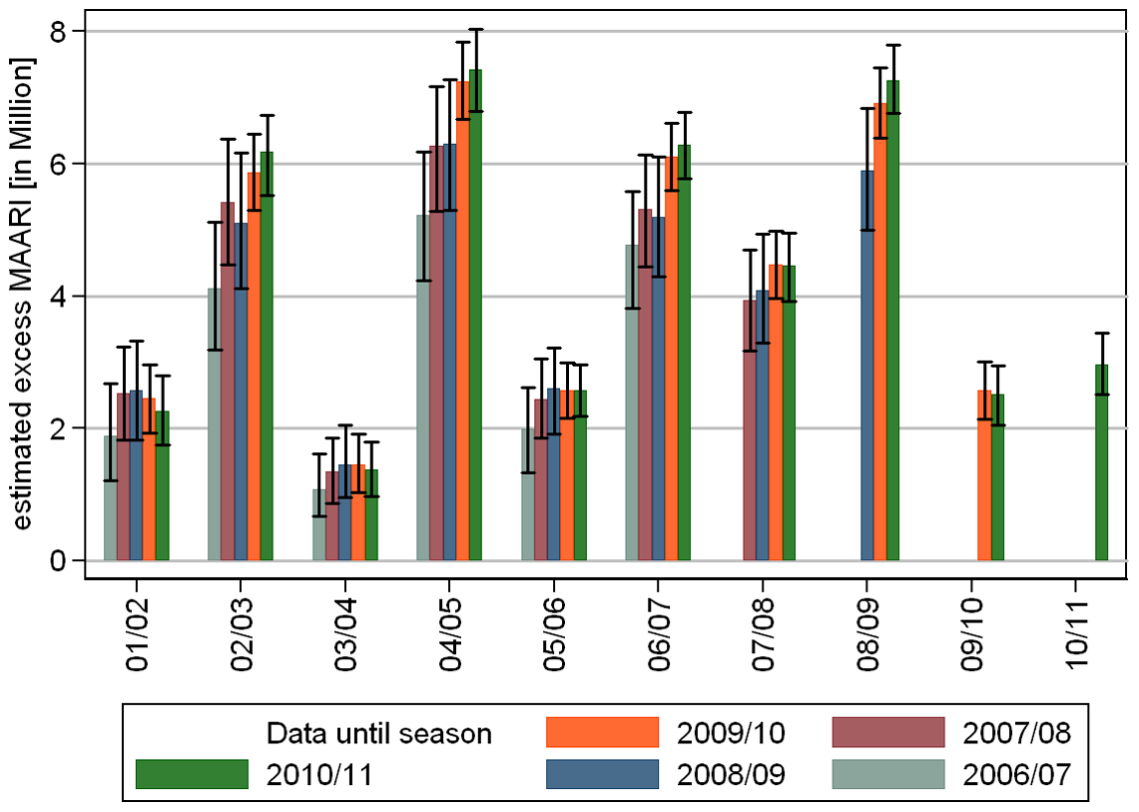
(Sensitivity analysis 2) Comparison of estimated total excess MAARI during epidemic periods depending on data of more seasons being available starting from 2006/07 to estimate the MAARI baseline.

**Figure 9:**
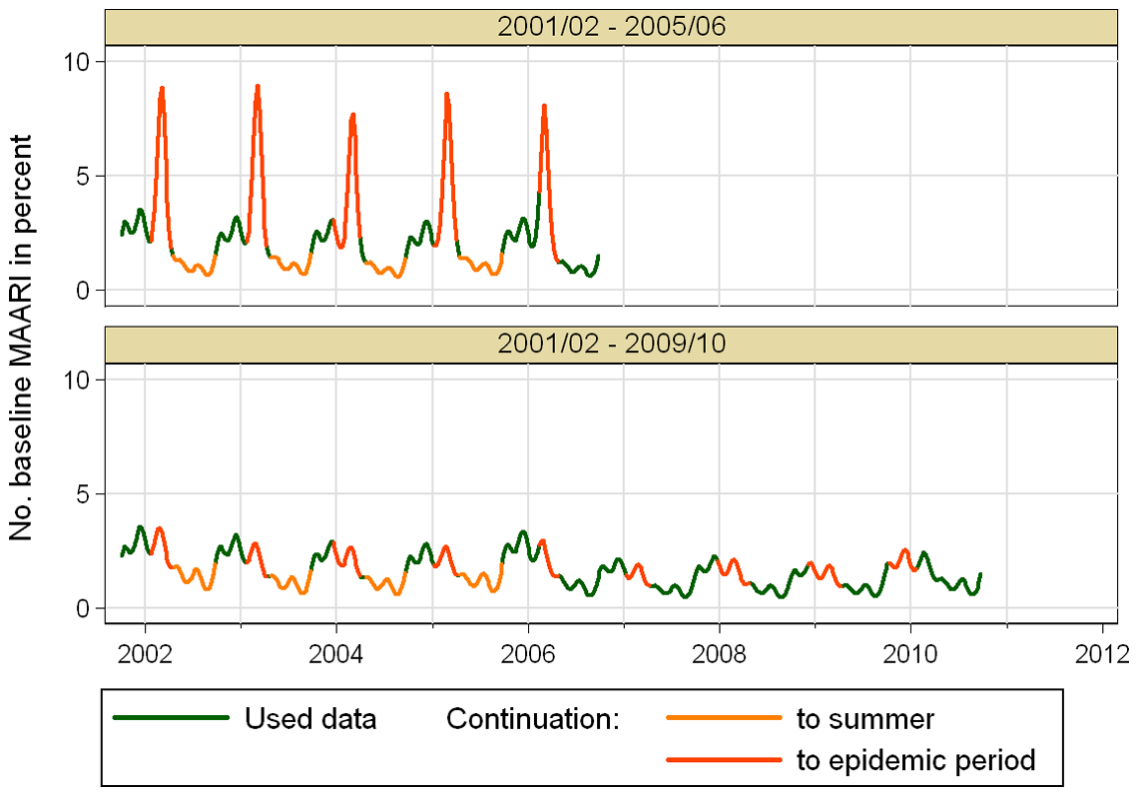
(Sensitivity analysis 2) Comparison of estimated MAARI baseline for 5–14 year old children in the southern region depending on the data set used.

The second sensitivity analysis starts with a data set including seasons 2001/02 to 2006/07, that was then step by step enlarged up to the whole range from 2001/02 to 2010/11. The model selection step resulted always in 5 overtones to the annual oscillation as well as oscillations with periods of up to half the length of the examination period. The polynomial trend had degree 3 for the data set that included season 2008/09, and degree 2 in all other cases. Comparing the estimated total excess MAARI showed larger deviations, see Figure 8. These deviations can be explained with the fact, that a stable estimation of the baseline needs sufficiently complete data. One point is that the first 4 seasons from 2001/02 to 2004/05 did not include a summer surveillance, which is particularly important for the estimation of the trend of the general consultation behavior. Even more important, omitting the data from the year of the 2009 pandemic influenza, leads to a data set in which only season 2005/06 has data during the calendar weeks 4 to 6 of the year allowing an artificially high peak in the baseline as shown in Figure 9 for the age group of 5–14 year old children in the southern region.

### Prospective estimation of excess MAARI

Since a stable estimation of the baseline necessitates the data from season 2006/07 up to 2009/10, we were able to test the prospective estimation of excess MAARI only for the epidemic period in season 2010/11.

A possible reporting delay was not of relevance, since in the seasons 2009/10 and 2010/11 all in all 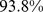 of the reports of AGI physicians were in time, i.e. have been reported in the week directly after MAARI occurred, whereas 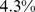 had a reporting delay of two weeks and 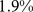 of more than two weeks.

**Figure 10:**
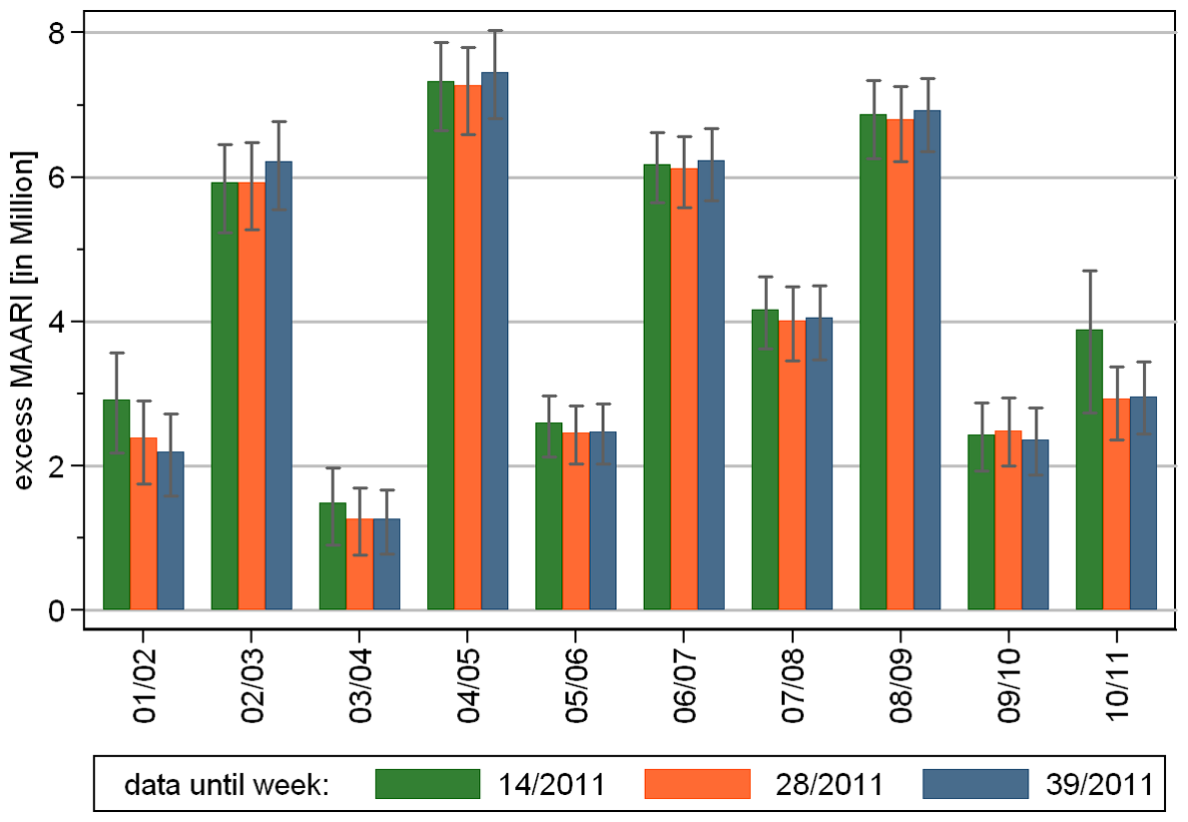
Prospective estimation of total excess MAARI: Changes after end of epidemic period depending on the data set.

We used the data set from season 2001/02 up to the last week of the epidemic period in season 2010/11 (week 14/2011) and compared the estimated total excess MAARI with the respective estimates given the data up to week 28/2011 and up to the end of the season in week 39/2011. The results are shown in Figure 10. Addition of new data, that contributed to the baseline, changed the estimated total excess MAARI only within the confidence limits. There was no clear trend over the years. Hence, at least for the epidemic period in season 2010/11 our approach was able to estimate the excess MAARI prospectively.

## Discussion

Using German syndromic surveillance data, we have developed a novel approach to describe the seasonal variation of MAARI and estimate the excess MAARI attributable to influenza retrospectively and prospectively from sentinel data. We defined epidemic periods for influenza independently of the syndromic data based on virological data. While we did not make assumptions about the shape of the influenza waves, we modeled the MAARI baseline taking into account geographic variation and different contribution by age groups. Moreover, changing amplitudes of the baseline over the years were incorporated in a flexible way. A simulation of the predictive distribution of the model for the baseline allowed us to quantify the uncertainty and also to accumulate the results in a straight forward way. The model was shown to be robust concerning the amount of available data.

Estimating the excess MAARI during an ongoing influenza epidemic leads to an estimate of the MAARI attributable to influenza up to the current week. Since the excess might be negative in the following weeks this estimate might decrease again in the future until the end of the epidemic period. Also, the addition of data in weeks after the end of an epidemic period may change the estimated MAARI baseline and thus change the estimated MAARI excesses retrospectively to a certain extent. Our sensitivity analysis has shown, however, that the model was able to estimate the total excess MAARI in 2011 already in the last week of the epidemic period with sufficient precision. Choosing four regions in Germany allowed us to see some spatial variation – on the other hand the regions had to be large enough to allow a stable projection of the MAARI data. In countries with a health system that defines a fixed catchment population for each physician, the same model might be able to describe a greater number of different region. In that case it might be advantageous to use a mixed Poisson model and treat region as a random variable.

### Comparison to other approaches

In contrast to Germany most European countries and the US monitor influenza like illness (ILI) to describe the epidemiologic activity of influenza. Collecting ARI results in a more complete picture of the influenza activity, or – stated differently – of the total burden in the population. First, approximately one fourth [1] to one third [2] of influenza present as ARI, not ILI. This proportion can also differ according to the subtypes of influenza viruses circulating. Moreover, the proportion of ARI that are truly influenza cases is very different than that for ILI and cannot be approximated by a simple factor. As a consequence, we cannot use the approach, that Goldstein et al. [3] have chosen, since we did not measure the number of medically attended ILI cases in Germany. A more direct estimation of the MAARI caused by influenza using virological results of samples from MAARI cases is in principle feasible, but this approach would be subject to several practical limitations. The number of samples that need to be taken to collect a relevant number of characterizable viruses in the beginning and end of the influenza season would need to be increased substantially, because MAARI is less specific compared to ILI and the positivity rate would be smaller. Moreover, the positivity rate depends on age group and to a smaller extent also on region, hence only representative samples of MAARI in different age groups (and potentially regions) would allow reasonably precise estimates of the burden of disease due to influenza.

Our method is a variant of Serfling's method [4]–[6], which is used in particular to estimate excess mortality associated with influenza circulation [7]. The main innovation is that we consider additional oscillations with periods of 2 and more years to account for changing heights of the baseline over the years. These oscillations in combination with a polynomial trend allow a very flexible fit of the baseline, see Figure 2.

Additional to the yearly oscillations we use oscillations with higher frequencies to keep the shape of the yearly pattern more flexible. In contrast to [6] the inclusion oscillations with more than one period per year did not lead e.g. to a semiannual pattern, but to a more complicated shape of the annual pattern.

Jansen et al. [8] investigated six different methods to estimate the excess MAARI and recommended to use the rate-difference method to estimate the excess MAARI attributable to influenza. However, this method has some drawbacks that we were able to resolve with our model. Namely, the excess MAARI estimated by the rate-difference method heavily depends on the chosen reference period outside the PIC. Jansen et al. propose two different reference periods, firstly the periseasonal model, where only influenza free weeks in the ‘winter season’ (between calendar week 40 of one year and week 20 of the next year) are considered and secondly the summer model, where all influenza free weeks were used to estimate the weekly MAARI baseline rate. This leads to huge differences in the estimated number of excess MAARI, which are also depending on season and age group. Even the smaller estimate (from the periseasonal model) could lead to an overestimation of the excess MAARI, when MAARI caused by other reasons than influenza or RSV peaks during an influenza active period. A similar drawback is common to all methods that avoid the estimation of a time varying baseline, since they have to estimate the amount of MAARI not related to influenza by some constant threshold [9].

A drawback in our model is that we can only use influenza-free weeks to fit the baseline model. The assumption that the continuation of the MAARI baseline describes the course of MAARI other than influenza also during influenza epidemic periods is in line with the observed course when the influenza epidemic period is shifted to different times of the year. In particular, the pandemic wave in 2009/10 ended already in calendar week 4. In addition, the assumption seems reasonable, because the secular trend and oscillations with at most 2 periods per year are stable enough to bridge the epidemic periods.

A way to include all data in the estimation of the baseline is to include a variable for the influenza activity in the model. For example the number of laboratory confirmed cases of influenza might describe the shape of the influenza wave consistently over time [10]–[12]. Yang et al. [13] assume that weekly proportions of positive specimens is a consistent measure of the virus activity and incorporated these proportions for influenza, RSV, adenovirus and parainfluenza in a model together with natural cubic spline smoothing functions of time, weekly average temperature and relative humidity.

In a setting where only the influenza caused MAARI should be estimated, we preferred the Serfling like approach, since the excluded periods were manageable and we did not have to assume a constant probability over time for diagnosis and reporting in the national mandatory reporting system or the virological surveillance of the NRCI.

### Discussion of the results

Our results show very nicely the age dependency of both background MAARI and excess MAARI attributed to influenza. Lower age groups experienced a substantially higher proportion of the MAARI baseline as well as excess MAARI compared to older age groups. It was confirmed convincingly that during the pandemic season 2009/10 the age group (5–14) was the one that was most affected – 9.6% (95%-CI 7.4%–12.0%) having consulted a physician, whereas the age group of infants (0–4) was less affected. In the seasons before 2005/06 the school aged children (5–14) were the most affected age group, but since then children (0–4) were most affected with the exception of the pandemic. This is also supported by data from the EPIA project published by Paget et al. [12].

It was intriguing that the MAARI baseline (Figure 2) and the estimated excess MAARI (data not shown) for children was higher in the Eastern German region compared to the other three German regions. This region matches with the former German Democratic Republic (GDR). For young adults aged 15–34 this effect was also visible during the first years of analysis, but seem to wane in the later years. These results are not easy to interpret; there are social economic differences between east and west Germany [14], [15], but the children and young adults of the eastern Germany do not seem to be in general more prone to become ill [16]. Eastern citizens may therefore simply be more inclined to seek health care when ill with the same disease. This is in line with higher vaccination rates for influenza in eastern Germany, see [17].

### Limitations

The flexibility on our model relies on consistent sentinel data over 10 years and necessitates at least 5 years of data throughout the year as we showed in the sensitivity analysis illustrated by Figures 8 and 9.

In general epidemic periods may be different in different regions and also for different age groups. Due to limited virological data we were not able to take these differences into account, but estimated only a single epidemic period valid for all strata.

To describe this baseline we used only data on the reported MAARI outside of epidemic periods. Hence, the method is only applicable when these periods are large enough to allow a stable estimation. In particular, a surveillance throughout the whole year is necessary. Moreover, it is important to have at least some seasons with data in a given calendar week. If a block of several calendar weeks is excluded in all seasons this might lead to artifacts as described in the second sensitivity analysis in section.

We did not adjust in our model for auto-correlation in the data, because we did not see an easy way to do it and also doubt that the point estimates would significantly change under this adjustment. Moreover, the coverage of the predictive intervals seems to be sufficient, see Figures S1 and S2 in the supporting information.

Generally our method can adjust for different MAARI background in the different seasons including a secular trend, but is less capable to reflect abrupt and substantial changes in the general consultation behavior or the surveillance system. Also interference between different respiratory viruses can not be described by our model.

The method seems to work well for a single pathogen, but there is no straight forward generalization for more than one pathogen, if the periods, where these pathogens circulate, are overlapping.

### Conclusions

In conclusion, we have devised a cyclical regression model that is capable to estimate the overall burden of influenza-associated MAARI. The model is robust, when at least 5 years of consistent sentinel surveillance data throughout the whole year are available. It takes into account the modifying roles of age, region and secular trends, and at least in the season 2010/11 it was able to calculate excess MAARI also during an evolving influenza epidemic. With additional data collected the model can be used as a valid starting point for case-fatality and economic impact estimations.

## Materials and Methods

### Data sources and definitions

In Germany, a syndromic, physician-based surveillance system for influenza (‘Arbeitsgemeinschaft Influenza’, AGI [18]) was established and began to function in 1993. The syndromic surveillance system relies on voluntarily participating private physicians in primary care (general practitioners (GP's), internists in primary care and pediatricians) who report aggregated numbers of MAARI among their patients.

In the following we consider ‘seasons’ from calendar week 40 up to week 39 of the following year. Until 2005, data were collected only in the ‘winter season’, i.e. from calendar week 40 to week 15. We will use the available data on MAARI in the time period between week 40/2001 up to week 39/2011, covering 10 seasons. In this period the reporting AGI physicians represent about 1% of all GP`s and about 2% of all pediatricians selected to be geographically representative of all registered primary care physicians in Germany.

To account for different regional dynamics we grouped the federal states into four disjoint regions: Southern(Bavaria and Baden-Württemberg), Western (Hesse, North Rhine-Westphalia, Rhineland-Palatinate, Saarland), Northern (Bremen, Hamburg, Lower Saxony, Schleswig-Holstein), Eastern (Berlin, Brandenburg, Mecklenburg-West Pomerania, Saxony, Saxony-Anhalt, Thuringia).

ARI was defined as acute pharyngitis, bronchitis or pneumonia with or without fever. Physicians reported weekly the number of MAARI for five age groups: (0–4), (5–14), (15–34), (35–59) and (60+) years old, as well as the total number of outpatient visits.

Virological surveillance was done with a sub-sample of about twenty percent of AGI physicians, who were instructed to take samples of the upper respiratory tract, e.g. nasal swabs, of a weekly number of 3–5 patients with influenza-like illness (ILI). Influenza-like illness was defined as an acute respiratory illness with fever and [cough or sore throat]. Physicians sent these samples to the National Reference Center for Influenza (NRCI). To define epidemic periods of influenza we sorted the samples by the calendar week, when they were taken (preferably) or received at NRCI. For each calendar week we calculated the positivity rate, i.e. the proportion of samples that were tested positive for influenza virus by PCR. The start of an epidemic period was defined as the first of two consecutive weeks, in which the lower 95%-confidence limit of the positivity rate was at least 10%. The end of the epidemic period was determined by the week that precedes the first two consecutive weeks, in which the lower 95%-confidence limit of the positivity rate drops below 10%.

### Estimation of excess MAARI

After projecting the sentinel data on MAARI to the German regions for each age group as described in the supporting information, we estimated excess MAARI by the following four steps:

1. We determined epidemic periods using the virological data as explained in section 0.
2. We estimated a MAARI baseline outside of the epidemic periods using the frequency analytic regression model described in subsection. The calendar weeks around the turn of the year (calendar week 52 and the following week) were excluded, because the number of MAARI is then regularly much lower and would distort the model.
3. We assumed that the continuation of the MAARI baseline to the epidemic periods is a valid description of MAARI not related to influenza.
4. We estimated the weekly excess MAARI as differences between the projected number of MAARI and the MAARI baseline. Excesses around the turn of the year were not estimated (see above). To estimate prediction intervals to the baseline we used a parametric bootstrap. Hence, we drew 1000 realizations of the baseline using the predictive distribution of the negative binomial model and, independently, of the projected weekly number of MAARI using independent normal distributions.

The cumulative excess for an epidemic period was defined as the sum of the weekly excess MAARI in a particular age group and region. Negative and positive weekly excesses were added up during the period inside the same region and age group. Negative weekly excesses were expected, since the MAARI baseline is smoother than the projected number of MAARI. In some cases it can be interpreted as the result of a harvesting effect, since only the first visit of a patient due to an ARI is documented in AGI system.

If the cumulative excess of an epidemic period was negative for a particular age group and region, we concluded that the number of MAARI caused by influenza can not be estimated with our method in that stratum. We defined the total (cumulative positive) excess of an epidemic period as the sum of the positive cumulative excesses. Additionally, the total negative excess was defined as the sum of the negative cumulative excesses in an epidemic period. It served as a control measures for the validity of the MAARI baseline. Cumulative and total excesses were analogously also calculated for the non-epidemic periods.

### Statistical model

Since the number of MAARI is a positive integer we used a regression model for counts. To adjust for overdispersion we chose a mean dispersion negative binomial regression model stratified by region and age group. The total population of the regions and age groups was used as offset in the model.

Our model for the MAARI baseline is a cyclic regression model similar to the model described by Serfling [4]. Additional to sine and cosine functions with a yearly period, we included faster oscillations with 2 or more periods per year to achieve a more realistic yearly pattern and slower oscillations with periods of 2 or more years to adjust for changing heights of the annual waves. Finally, we allowed a polynomial trend in time with a degree less or equal to 3.

In a model selection step we decided which oscillations were included in the model and how the polynomial trend was modeled. To find a parsimonious model with only the most important influences we aggregated the data over the regions and used a likelihood ratio test with significance level 1% for model comparison. Age group was used as a categorical variable. We included oscillations with periods of 1 up to 5 years (half the length of the examination period). For the secular trend we used – starting with a linear trend – sequentially polynomials with degree less or equal to 3. Oscillations with up to 6 periods per year were included sequentially. Interaction terms of age group with time dependent variables were allowed. Since oscillations in the model may have an effect on the estimated trend we iteratively repeated these steps until we found a stable model.

Having selected the general structure of the model we used this model to fit the estimated number of MAARI in the different regions and age groups.

Summing up, we described the MAARI in region 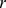 and age group 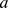 with a mean dispersion negative binomial distribution with expected value 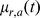 and shape parameter 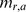. This results in a variance 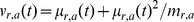. The expected value is given as
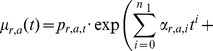


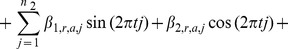


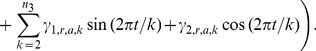
(1)Here, the parameters 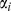 describe the polynomial trend in time and the 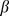
's and 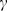
's are the amplitudes of the respective oscillations. The numbers 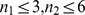 and 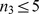 are determined as described above. 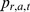 denotes the population of age group 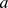 in region 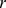 and time 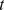, which is updated once a year at the start of the season.

This stratified model is equivalent to a generalized mean dispersion negative binomial regression model, where the dispersion parameter is allowed to depend on age group and region and their interaction term. In this generalized model we investigated the interactions between age group, region and the time dependent variables to see whether the differences were significant. In the full generalized model the shape parameter and the expected value are given by
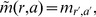
(2)

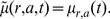
(3)


In case that a particular interaction with region or age group would not significantly improve the model, this interaction could be neglected. In consequence, the respective 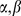 or 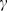 parameter would then not depend on region or age group, respectively.

### Sensitivity analysis

It may make a difference which seasons were used to construct the MAARI baseline. We therefore performed sensitivity analyses, in which we reduced the number of seasons to built the baseline. First, we neglected sequentially early seasons and observed the effect on the estimated total excess MAARI, then we started from season 2006/07 and checked how the estimates changed, when data of newer seasons were added.

All estimations including the calculations of prediction intervals of the generalized negative binomial regression model (command *gnbreg*) were done using the statistical software package Stata 12 (STATA Corporation, College Station, TX, USA).

### Prospective estimation of excess MAARI

Note that the beginning and the end of an epidemic period can by definition only be detected with a delay of two weeks (see section). During an epidemic period the MAARI baseline stays the same, because it is fitted only to data outside the epidemic periods. After the epidemic period the MAARI baseline is updated, because new data was added to its estimation. Consequently, also the estimated excess MAARI may then retrospectively change again.

In the course of an epidemic period the current cumulative excess MAARI can be estimated as the sum of the weekly excess. If it is negative, we conclude – similar to the retrospective interpretation – that we can not give an estimate of the current number of MAARI caused by influenza in that stratum. The total (cumulative positive) excess is then again defined as the sum of the positive cumulative excesses.

For seasons that allowed a stable estimation of the MAARI baseline with data from earlier seasons, we compared the estimate of the total excess MAARI at the end of the epidemic period with the one at the end of the season and an intermediate estimation.

## Supporting Information

Figure S1 **Observed MAARI (darkgreen) with 95% confidence interval (green) and estimated MAARI baseline (red) with 95% prediction interval (dotted red line) in different regions starting from season 2006/07, vertical lines indicate beginning and end of PICs; (A) age 0–4 years; (B) age 5–14 years; (C) age 15–34 years.**
(TIF)

Figure S2 **Observed MAARI (darkgreen) with 95% confidence interval (green) and estimated MAARI baseline (red) with 95% prediction interval (dotted red line) in different regions starting from season 2006/07, vertical lines indicate beginning and end of PICs; (A) age 35–59 years; (B) age 60+ years.**
(TIF)

File S1 **The file contains the following three sections: Practices and physicians; Estimation of MAARI; Projected MAARI and the MAARI baseline.**
(PDF)

## References

[1] Carrat F, Vergu E, Ferguson NM, Lemaitre M, Cauchemez S, et al (2008) Time lines of infection and disease in human influenza: a review of volunteer challenge studies. Am J Epidemiol 167: 775–785. 18230677 10.1093/aje/kwm375

[2] Suess T, Buchholz U, Dupke S, Grunow R, an der Heiden M, et al (2010) Shedding and transmission of novel influenza virus a/h1n1 infection in households–germany, 2009. Am J Epidemiol 171: 1157–1164. 20439308 10.1093/aje/kwq071

[3] Goldstein E, Viboud C, Charu V, Lipsitch M (2012) Improving the estimation of influenza-related mortality over a seasonal baseline. Epidemiology 23: 829–838. 22992574 10.1097/EDE.0b013e31826c2dda PMC3516362

[4] Sering RE (1963) Methods for current statistical analysis of excess pneumonia-influenza deaths. Public Health Rep 78: 494–506. 19316455 PMC1915276

[5] Simonsen L, Reichert TA, Viboud C, Blackwelder WC, Taylor RJ, et al (2005) Impact of influenza vaccination on seasonal mortality in the us elderly population. Arch Intern Med 165: 265–272. 15710788 10.1001/archinte.165.3.265

[6] Yang L, Wong CM, Chan KP, Chau PYK, Ou CQ, et al (2009) Seasonal effects of influenza on mortality in a subtropical city. BMC Infect Dis 9: 133. 19698116 10.1186/1471-2334-9-133 PMC2739210

[7] Thompson WW, Comanor L, Shay DK (2006) Epidemiology of seasonal influenza: use of surveillance data and statistical models to estimate the burden of disease. J Infect Dis 194 Suppl 2S82–S91. 17163394 10.1086/507558

[8] Jansen AGSC, Sanders EAM, Wallinga J, Groen EJ, van Loon AM, et al (2008) Rate-difference method proved satisfactory in estimating the influenza burden in primary care visits. J Clin Epidemiol 61: 803–812. 18495428 10.1016/j.jclinepi.2007.08.017

[9] Alonso TV, Alonsoa JEL, de Lejarazub RO, Prez MG (2004) Modelling influenza epidemic – can we detect the beginning and predict the intensity and duration? International Congress Series 1263: 281–283.

[10] Pitman RJ, Melegaro A, Gelb D, Siddiqui MR, Gay NJ, et al (2007) Assessing the burden of influenza and other respiratory infections in england and wales. J Infect 54: 530–538. 17097147 10.1016/j.jinf.2006.09.017

[11] Reed C, Angulo FJ, Swerdlow DL, Lipsitch M, Meltzer MI, et al (2009) Estimates of the prevalence of pandemic (h1n1) 2009, united states, april-july 2009. Emerg Infect Dis 15: 2004–2007. 19961687 10.3201/eid1512.091413 PMC3375879

[12] Paget WJ, Balderston C, Casas I, Donker G, Edelman L, et al (2010) Assessing the burden of paediatric influenza in europe: the european paediatric influenza analysis (epia) project. Eur J Pediatr 169: 997–1008. 20229049 10.1007/s00431-010-1164-0 PMC2890072

[13] Yang L, Chiu SS, Chan KP, Chan KH, Wong WHS, et al (2011) Validation of statistical models for estimating hospitalization associated with influenza and other respiratory viruses. PLoS One 6: e17882. 21412433 10.1371/journal.pone.0017882 PMC3055891

[14] Gray L, Merlo J, Mindell J, Hallqvist J, Tafforeau J, et al (2012) International differences in self-reported health measures in 33 major metropolitan areas in europe. Eur J Public Health 22: 40–47. 21148178 10.1093/eurpub/ckq170 PMC3265749

[15] Voigtländer S, Berger U, Razum O (2010) Increasing regional disparities in living conditions in germany and their role in the explanation of health inequalities. Gesundheitswesen 72: 301 308. 10.1055/s-0029-1233487 19662593

[16] Kamtsiuris P, Atzpodien K, Ellert U, Schlack R, Schlaud M (2007) Prvalenz von somatischen erkrankungen bei kindern und jugendlichen in deutschland. Bundesgesundheitsblatt – Gesundheitsforschung – Gesundheitsschutz 50: 686–700. 17514453 10.1007/s00103-007-0230-x

[17] Wiese-Posselt M, Leitmeyer K, Hamouda O, Bocter N, Zllner I, et al (2006) Influenza vaccination coverage in adults belonging to defined target groups, germany, 2003/2004. Vaccine 24: 2560–2566. 16414160 10.1016/j.vaccine.2005.12.020

[18] Arbeitsgemeinschaft Influenza (2012) Bericht zur Epidemiologie der Influenza in Deutschland Saison 2011/12. Robert Koch-Institut. URL Available: http://influenza.rki.de/Saisonberichte/2011.pdf. Accessed 2013 April 26.

