# Correction: Estimates of Excess Medically Attended Acute Respiratory Infections in Periods of Seasonal and Pandemic Influenza in Germany from 2001/02 to 2010/11

**DOI:** 10.1371/annotation/e469edc7-92f6-4126-9be1-79133e14a453

**Published:** 2014-01-02

**Authors:** Matthias an der Heiden, Karla Köpke, Silke Buda, Udo Buchholz, Walter Haas

The name of the first author, Matthias an der Heiden, was incorrectly given in the Citation. The correct Citation is: an der Heiden M, Köpke K, Buda S, Buchholz U, Haas W (2013) Estimates of Excess Medically Attended Acute Respiratory Infections in Periods of Seasonal and Pandemic Influenza in Germany from 2001/02 to 2010/11. PLoS ONE 8(7): e64593. doi:10.1371/journal.pone.0064593.

In addition, the first author's name was incorrectly represented in the Copyright Statement. The first sentence of the Copyright Statement should read: © 2013 an der Heiden et al.

In addition, there were multiple errors in the Results, Discussion and Materials and Methods. The second sentence of the first paragraph of the "Periods of influenza season" section of the Results is incorrect. The sentence should read: "The epidemic period began always shortly before or after the turn of the year, only in season 2005/06 it started in February, see Table 1."

The fourth sentence of the third paragraph of the "Limitations" section of the Discussion is incorrect. The sentence should read is: "If a block of several calendar weeks is excluded in all seasons this might lead to artifacts as described in the second sensitivity analysis in section Results, subsection Sensitivity analysis."

The sentence comprising point 1 of the first paragraph of the "Estimation of excess MAARI" section of the Materials and Methods is incorrect. The sentence should read: "We determined epidemic periods using the virological data as explained in subsection Data sources and definitions."

The sentence comprising point 2 of the first paragraph of the "Estimation of excess MAARI" section of the Materials and Methods is incorrect. The sentence should read: "We estimated a MAARI baseline outside of the epidemic periods using the frequency analytic regression model described in subsection Statistical model."

The first sentence of the first paragraph of the "Prospective estimation of excess MAARI" section of the Materials and Methods is incorrect. The sentence should read: "Note that the beginning and the end of an epidemic period can by definition only be detected with a delay of two weeks (see subsection Data sources and definitions)." 

