# Supplementary figures and images for: Estimates of Excess Medically Attended Acute Respiratory Infections in Periods of Seasonal and Pandemic Influenza in Germany from 2001/02 to 2010/11
Source: PLoS One. 2013 Jul 16;8(7):e64593. doi: 10.1371/journal.pone.0064593 (PMC3712969; doi:10.1371/journal.pone.0064593)

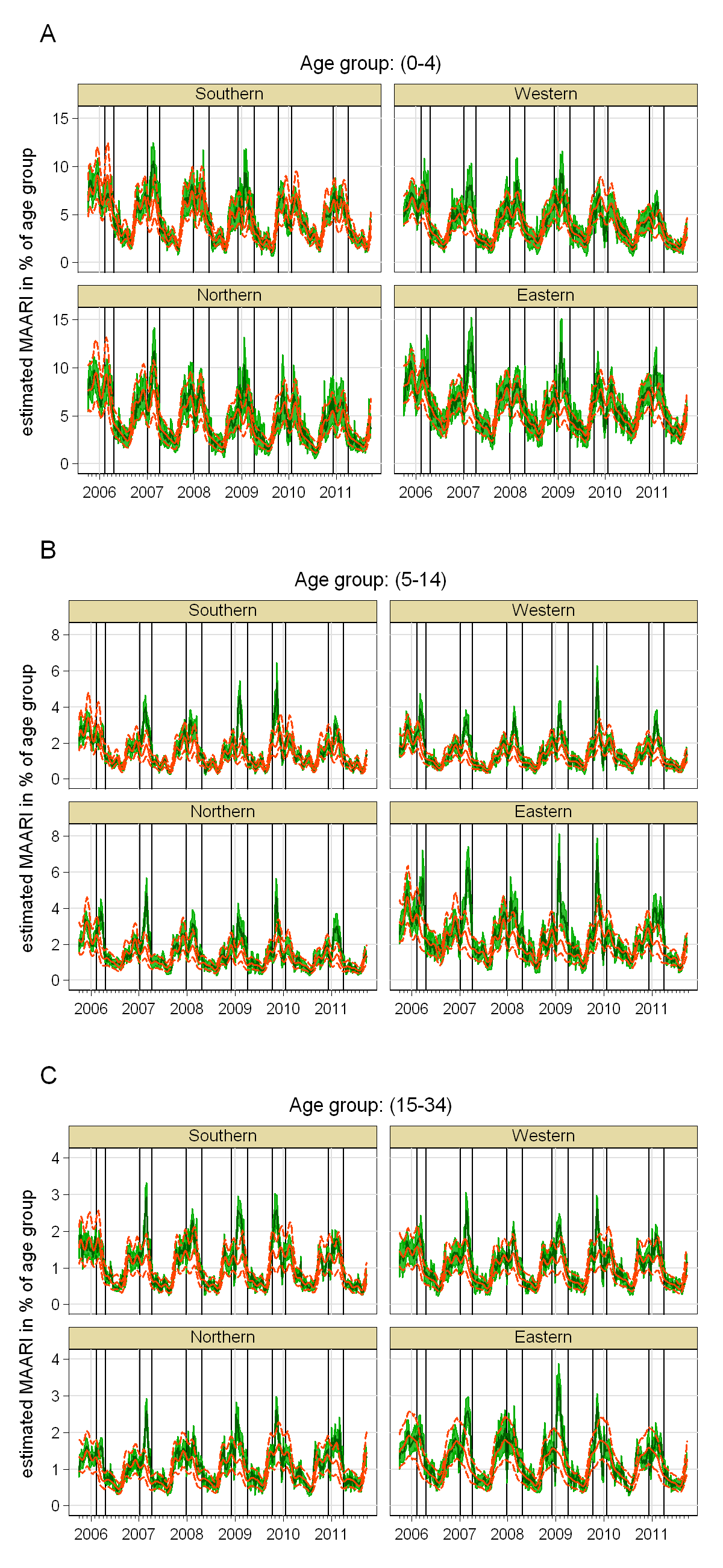

Supplement: Figure S1 — Observed MAARI (darkgreen) with 95% confidence interval (green) and estimated MAARI baseline (red) with 95% prediction interval (dotted red line) in different regions starting from season 2006/07, vertical lines indicate beginning and end of PICs; (A) age 0–4 years; (B) age 5–14 years; (C) age 15–34 years. (TIF) [file pone.0064593.s001.tif]

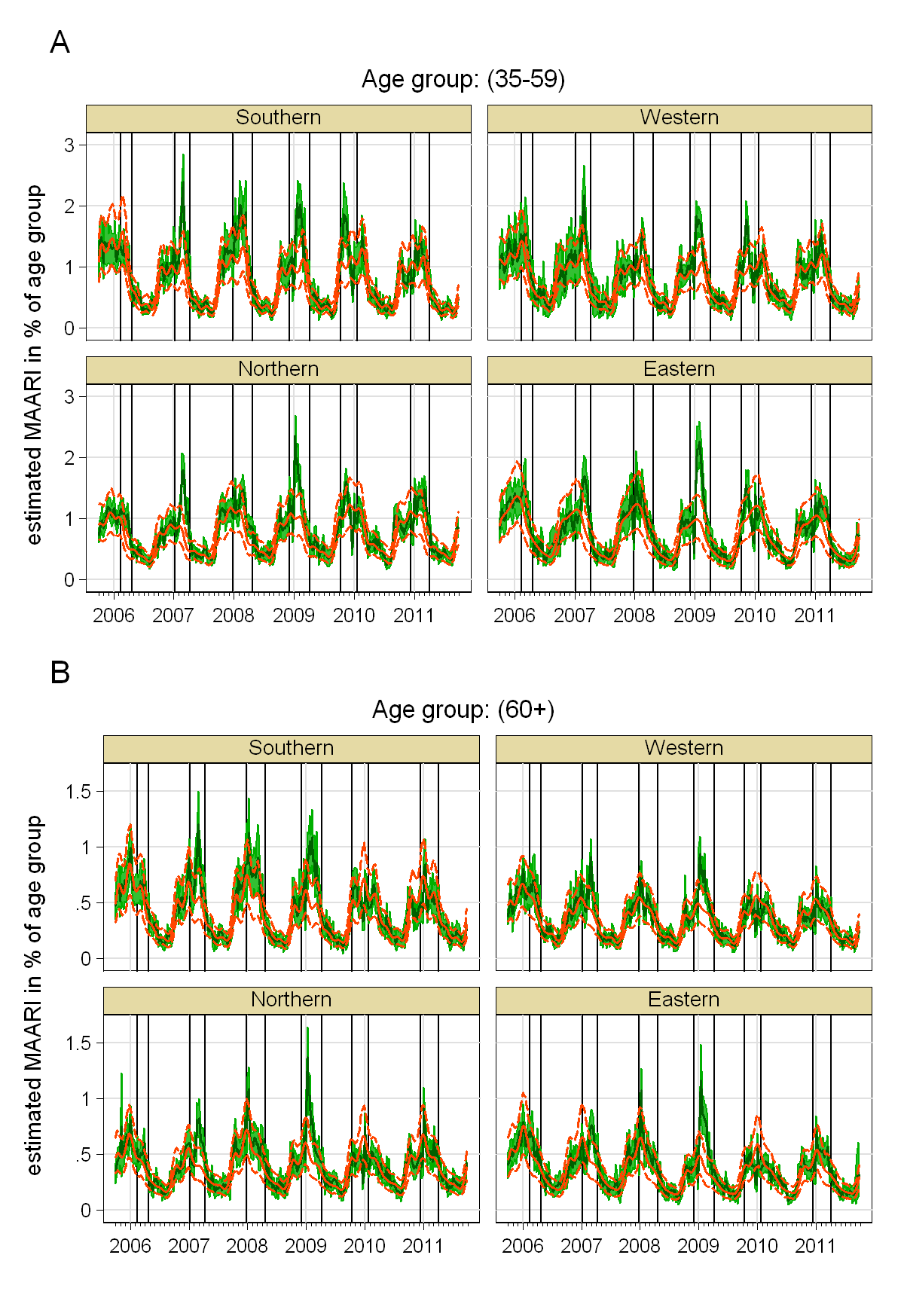

Supplement: Figure S2 — Observed MAARI (darkgreen) with 95% confidence interval (green) and estimated MAARI baseline (red) with 95% prediction interval (dotted red line) in different regions starting from season 2006/07, vertical lines indicate beginning and end of PICs; (A) age 35–59 years; (B) age 60+ years. (TIF) [file pone.0064593.s002.tif]
