# Supplementary material for: Estimates of Excess Medically Attended Acute Respiratory Infections in Periods of Seasonal and Pandemic Influenza in Germany from 2001/02 to 2010/11
Source: PLoS One. 2013 Jul 16;8(7):e64593. doi: 10.1371/journal.pone.0064593 (PMC3712969; doi:10.1371/journal.pone.0064593)
Supplement: File S1 — The file contains the following three sections: Practices and physicians; Estimation of MAARI; Projected MAARI and the MAARI baseline. (PDF) [file pone.0064593.s003.pdf]

1 Supplementary Information

1.1 Practices and physicians

The AGI sentinel system contacts practices whereas our analysis relies on physicians. Therefore, as a first step we convert the practice information into information about physicians. This is easy for practices with only one physician. For practices with more than one physician we proceed in the following way: if all physicians have the same specialty assume that each of these physicians have served the an equal proportion of the patients for the whole practice. In case that a pediatrician runs a practice together with a GP we assume that all patients younger than 15 year were served by the pediatrician, while all older patient were served by the GP. In case that an internist in primary care runs a practice together with a GP we assume that all patients younger than 15 year were served by the GP, while the internist and the GP serve an equal proportion of all patient older than 14.

1.2 Estimation of MAARI

Since there is no fixed catchment population for a physician due to the free choice of medical practitioner in Germany, there is no denominator for the number of MAARI of a single physician. We estimated therefore the total number of consultations in a region as the projection of the number of MAARI reported by AGI physicians to the total number of physicians in that region. This kind of estimation of MAARI on a population level was already described in [1]. The projection was done separately for pediatricians and general practitioners. For patients aged at least 15 years we also included internists as long as they are registered „primary care provider“ into the group of general practitioners. On the other hand we restricted the projection of the pediatricians to patients younger than 35.

We used data of the Association of Statutory Health Insurance Physicians to obtain the total number of physicians in region r with specialty s - either pediatrician or GP. In the age groups (15-34), (35-59) and (60+) the number of internists in primary care was added to the number of GP's. Hence, in the following this number is age dependent. The numbers of physicians were updated every year at the beginning of the season. The average number of physicians by specialty and region in Germany over the years 2001 to 2010 can be seen in Table S1.

We describe now how the number of MAARI was estimated for each age group and region: Let w_y be the total number of weeks in year y, that is $w_y = 53$ for the years 2004 and 2009 and $w_y = 52$ for all other years between 2001 and 2011. For each year $y \in \{2000, \ldots, 2010\}$ and calendar week $w \in \{1, \ldots, w_y\}$ we define

the time t by

$$t = y - 2000 + (w - 1)/w_y. \tag{1}$$

We denote by $P_{r,a,s,t}$ the set of AGI physicians in the region r with specialty s, who sent a report in the week given by t. We denote by $R_{i,a,t}$ the number of MAARI in age group a, that are reported by physician i in the week given by t.

We assume that the AGI physicians reported all cases of MAARI according to the case definition. Based on this assumption the $\{R_{i,a,t}, i \in P_{r,a,s,t}\}$ for fixed region r, specialty s and calendar week given by t can be treated as independent identically distributed random variables. Hence, we can estimate the mean number of MAARI of age group a per physician as

$$\langle R \rangle_{r,a,s,t} = \frac{\sum_{i \in P_{r,a,s,t}} R_{i,a,t}}{|P_{r,a,s,t}|}. \tag{2}$$

We assume that the consultation behavior of patients of AGI physicians is representative for the consultation behavior of all patients. Let $n_{r,a,s}$ be the total number of physicians of specialty s in region r relevant for age group a. Then we obtain the total number of MAARI, $M_{r,a,s,t}$, as a projection of the mean number of MAARI per physician to the corresponding total number of physicians (see Table S1)

$$M_{r,a,s,t} = n_{r,a,s} * \langle R \rangle_{r,a,s,t}. \tag{3}$$

The standard error of $M_{r,a,s,t}$ equals the standard error of the mean (2) multiplied by $n_{r,a,s}$.

The total number of MAARI of an age group is given by the sum of MAARI attended by pediatricians and those attended by GP's:

$$M_{r,a,t} = M_{r,a,\mathtt{ped},t} + M_{r,a,\mathtt{gp},t}. \tag{4}$$

Since the two summands are statistically independent of each other, the resulting standard error is

$$\sigma_{M_{r,a,t}} = \sqrt{\sigma^2_{M_{r,a,\mathtt{ped},t}} + \sigma^2_{M_{r,a,\mathtt{gp},t}}}. \tag{5}$$

Since the total number of consultations results from the estimation of a mean of independent identically distributed random variables $R_{i,a,t}, i \in P_{r,a,s,t}$, it is asymptotically normally distributed. The 95% confidence interval is then approximated by

$$M^{\pm}_{r,a,t} = M_{r,a,t} \pm \Phi^{-1}(.975) * \sigma_{M_{r,a,t}}, \tag{6}$$

where Φ denotes the cumulative distribution function of the normal distribution, in particular $\Phi^{-1}(.975) \approx 1.96$.

Let $p_{r,a,t}$ denote the population of age group a in region r and time t.

Then the MAARI incidence is given by

$$MI_{r,a,t} = \frac{M_{r,a,t}}{p_{r,a}}. \tag{7}$$

1.3 Projected MAARI and the MAARI baseline

The regional baselines together with their 95% upper and lower prediction limits and the projected number of MAARI for years with summer surveillance data are shown in Figures S1 and S2.

References

[1] Uphoff H, Stilianakis N (2000) Ein Ansatz zur bevölkerungsbezogenen Auswertung der deutschen Influenza-Sentineldaten. Bundesgesundheitsblatt - Gesundheitsforschung - Gesundheitsschutz 43: 796-801.

Tables

Region	pediatricians	GP's	internists in primary care
Southern	1621	13930	2830
Western	1948	13493	4335
Northern	883	6694	1671
Eastern	1362	8599	2289
Germany	5815	41716	11124

Table S1: Average number of physicians by specialty and region in Germany over the years 2001 to 2010.
